# Supplementary figures and images for: Extracellular domain, hinge, and transmembrane determinants affecting surface CD4 expression of a novel anti-HIV chimeric antigen receptor (CAR) construct
Source: PLoS One. 2024 Aug 12;19(8):e0293990. doi: 10.1371/journal.pone.0293990 (PMC11318886; doi:10.1371/journal.pone.0293990)

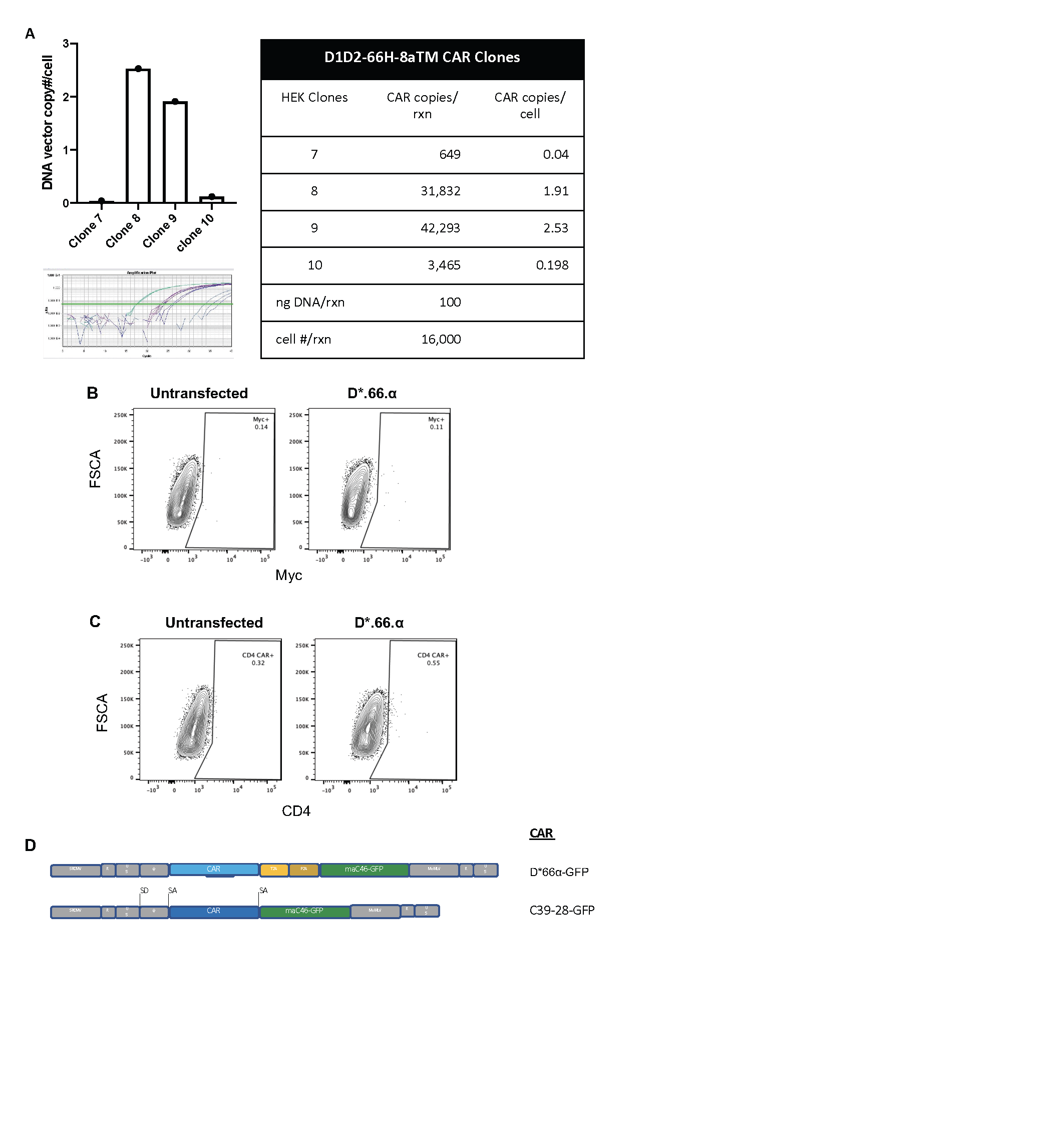

Supplement: S1 Fig — A. Histogram representing qPCR results and CAR DNA copy number in transduced HEK293 T cells. B. Representative flow plots showing surface detection of CAR by anti myc-tag antibody transfected HEK293T cells. C. Representative flow plots showing surface detection of CAR by anti-CD4 antibody in transfected HEK293T cells. D. Vector map representing the bicistronic GFP constructs. (TIFF) [file pone.0293990.s001.tiff]

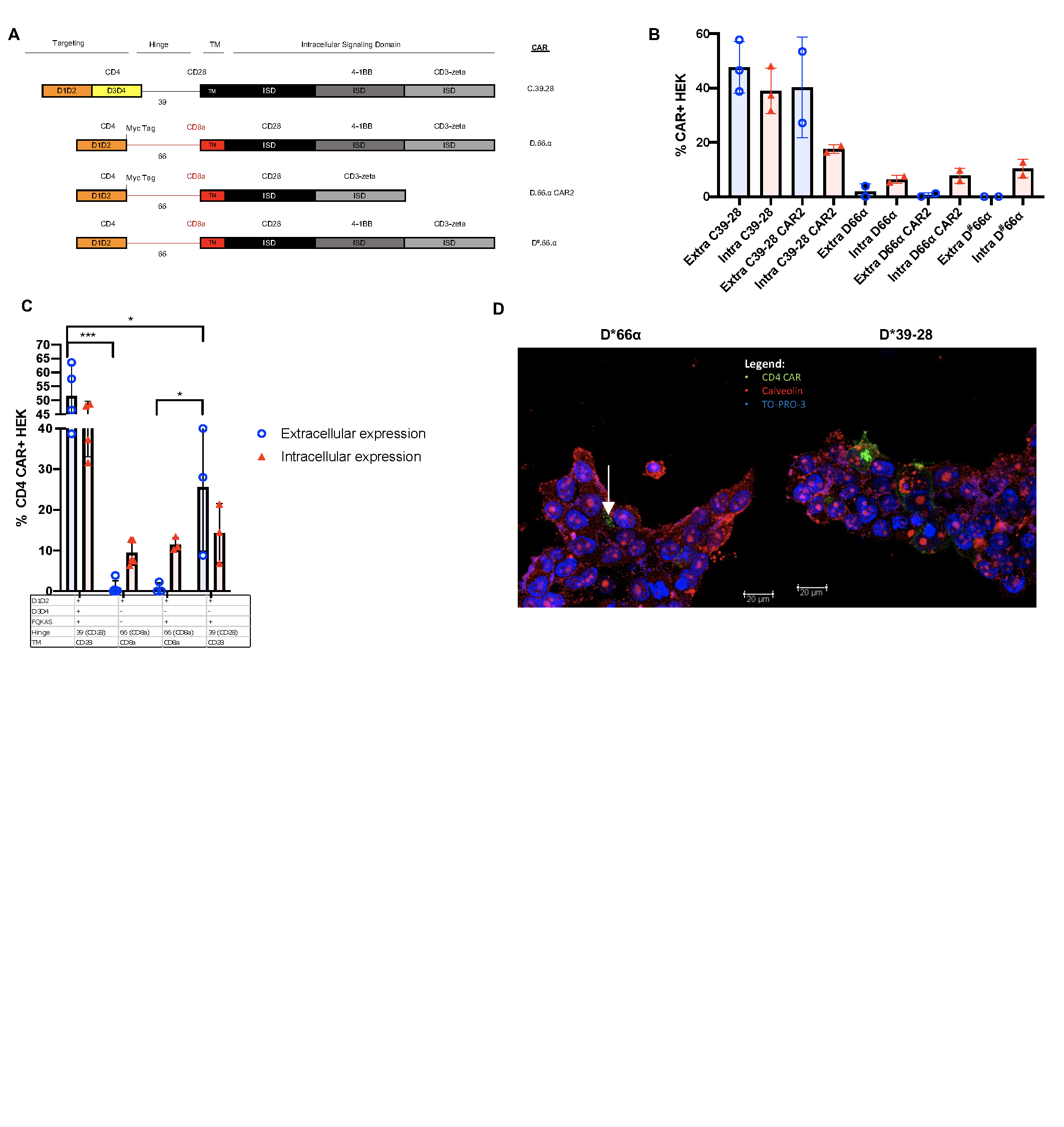

Supplement: S2 Fig — A. Schematic diagram representing the CAR vector maps for the constructs used in this study (along with D*.39.28 in Fig 1). B. Histogram representing the proportion of transfected HEK 293T cells expressing surface CD4 CAR and intracellular CD4 CAR for constructs with variation in ISD or Myc Tag. Positive CAR detection was observed on the surface of all C39-28-CARs but on none of the D66α CARs, suggesting ISD and Myc tag had no effects on CAR surface expression. C. Histogram representing the proportion of transfected HEK 293T cells expressing surface CD4 CAR and intracellular CD4 CAR for various CARs. Positive CAR detection was observed intracellularly but not on the cell surface of HEK293T cells transfected with both D66α and D*66α CARs. In addition, no significant difference in expression was detected across groups with or without the FQKAS motif (p = 0.911). Robust surface detection was observed in D*39–28 but not in D*66α (p = 0.01). No significant difference was observed in intracellular CAR detection between the two vectors (p = 0.359). Statistical analysis was done using unpaired parametric two-sample t-test. D. Images of CAR-transfected HEK 293T cells taken by confocal microscopy. Blue is TO-PRO-3, representing nuclear stain; green is CD4-CAR; and red is calveolin, representing the cell membrane. Differences in confocal surface CAR detection were observed, with D*66α lacking surface expression but not D*39–28. (TIFF) [file pone.0293990.s002.tiff]

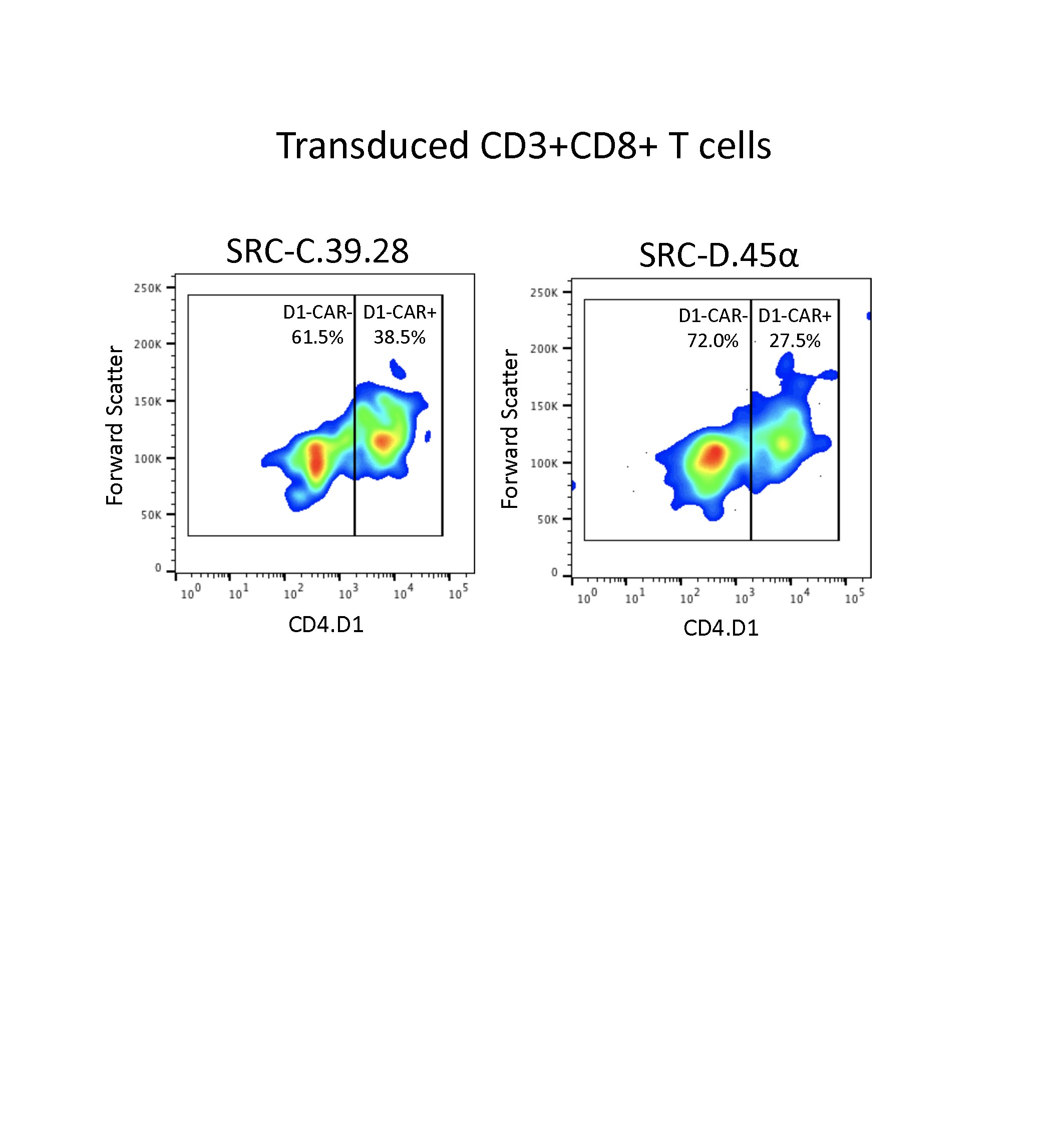

Supplement: S3 Fig — Rhesus PBMCs were stimulated with CD3/CD28 beads plus 100 IU/ml IL-2 and exposed to viral supernatant with Retronectin. Cells were expanded and stained for flow cytometry. CD3+CD8+ T cells were gate and CAR expression was assessed with a human specific CD4 antibody (clone RPA-T4). These data show that between 27%-38% of the T cells expressed the CAR. (TIFF) [file pone.0293990.s003.tiff]

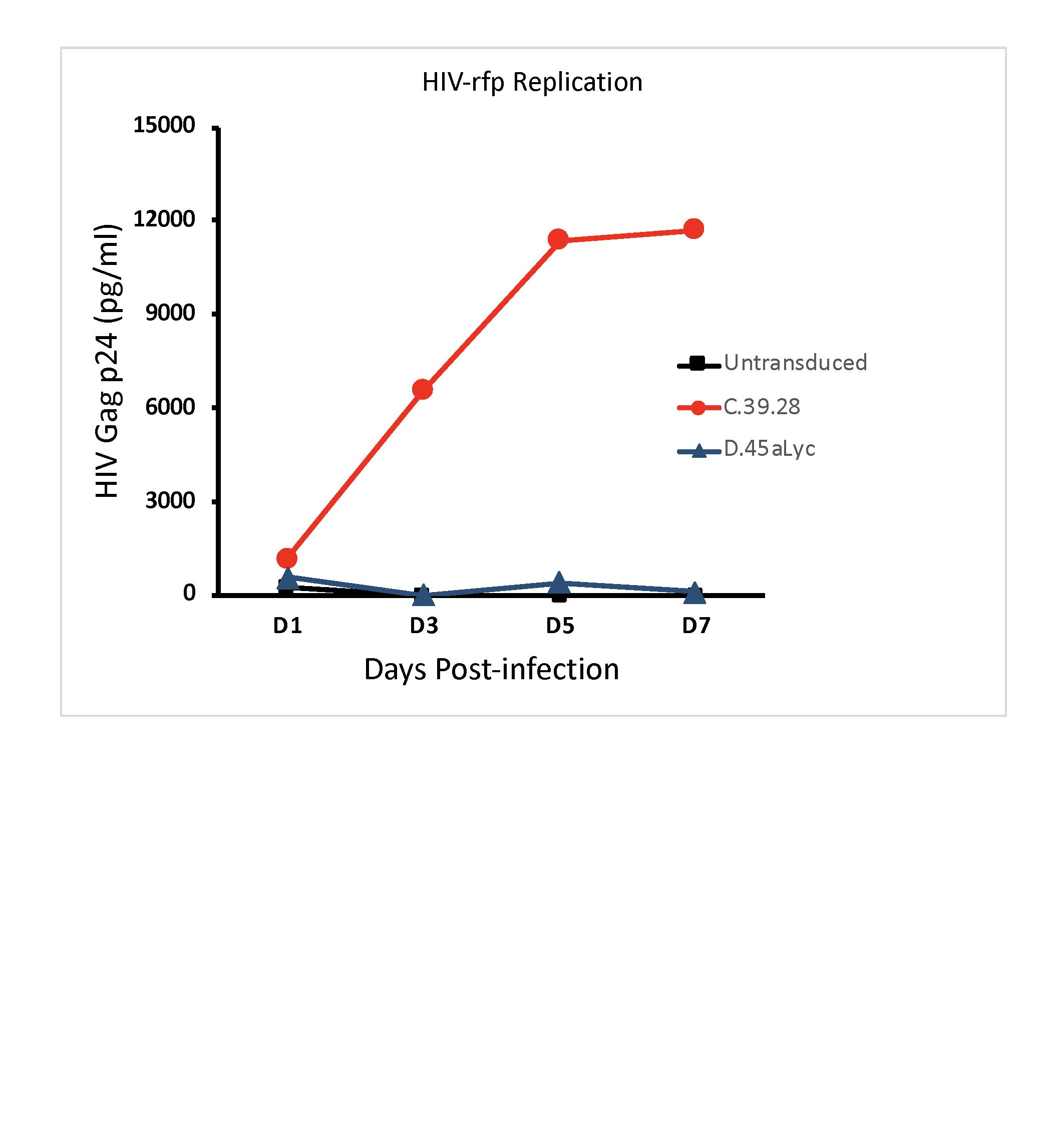

Supplement: S4 Fig — Line graph of HIV-1 viral replication as measured by HIV-1 p24 Gag production. HEK293T cells were untransduced or transduced with the full-length CD4-CAR vector (C.39.28) or the D1D2 truncated variant with 45 aa hinge and TM domain from CD8a including the LYC intracellular sequences (D.45.αLYC). Cells were challenged with HIV-rfp. After Day 1, Day 3, Day 5, and Day 7; culture supernatants were collected for analysis of p24 Gag production by ELISA. As expected, the untransduced HEK293T cells are not susceptible to HIV-1 infection. When HEK293T cells are expressing the full-length CD4 (C.39.28), they support HIV-1 viral replication; however, HEK293T cells expressing D1D2 with the CD8a hinge/TM domain (D.45.αLYC) do not support viral replication. (TIFF) [file pone.0293990.s004.tiff]
