# Supplementary material for: Extracellular domain, hinge, and transmembrane determinants affecting surface CD4 expression of a novel anti-HIV chimeric antigen receptor (CAR) construct
Source: PLoS One. 2024 Aug 12;19(8):e0293990. doi: 10.1371/journal.pone.0293990 (PMC11318886; doi:10.1371/journal.pone.0293990)
Supplement: S1 Table — Domains are color coordinated; signal peptide (yellow), D1D2 EC domain (light blue), D3D4 Ec domain (light gray), CD28 EC, TM, or IDC (Red), CD8a hinge (green); CD8a TM (bold black), 4-1BB ICD (green), CD3 zeta IDC (gray). Dots represent sequences identical to the C.39.28; hyphens represent sequence gaps. TM prediction of CD8 hinge, CD8 TM, CD28 ICD. (DOCX) [file pone.0293990.s005.docx]

Signal peptide

D1D2 CD4 EC domain

D3D4 CD4 EC domain

CD28 EC, TM, ICD

Hinge (CD8α)

**TM (CD8α)**

4-1BB ICD

CD3 zeta ICD

**. is matched identy**

**- is deleted amino acid**

C.39.28 1 MNRGVPFRHLLLVLQLALLPAATQGKKVVLGKKGDTVELTCTASQKKSIQFHWKNSNQIKILGNQGSFLTKGPSKLNDRADSRRSLWDQG 90

D*.39.28 1 .......................................................................................... 90

D*.45.α 1 .......................................................................................... 90

D*.45.αLYC 1 .......................................................................................... 90

D*.66.α 1 .......................................................................................... 90

D.66.α 1 .......................................................................................... 90

C.39.28 91 NFPLIIKNLKIEDSDTYICEVEDQKEEVQLLVFGLTANSDTHLLQGQSLTLTLESPPGSSPSVQCRSPRGKNIQGGKTLSVSQLELQDSG 180

D*.39.28 91 .......................................................................................... 180

D*.45.α 91 .......................................................................................... 180

D*.45.αLYC 91 .......................................................................................... 180

D*.66.α 91 .......................................................................................... 180

D.66.α 91 .......................................................................................... 180

C.39.28 181 TWTCTVLQNQKKVEFKIDIVVLAFQKASSIVYKKEGEQVEFSFPLAFTVEKLTGSGELWWQAERASSSKSWITFDLKNKEVSVKRVTQDP 270

D*.39.28 181 .......................FQKAS-------------------------------------------------------------- 211

D*.45.α 181 .......................FQKAS.G------------------------------------------------------------ 209

D*.45.αLYC 181 .......................FQKAS.G------------------------------------------------------------ 209

D*.66.α 181 .......................FQKAS-------------------------------------------------------------- 210

D.66.α 181 .......................------------------------------------------------------------------- 211

C.39.28 271 KLQMGKKLPLHLTLPQALPQYAGSGNLTLALEAKTGKLHQEVNLVVMRATQLQKNLTCEVWGPTSPKLMLSLKLENKEAKVSKREKAVWV 360

D*.39.28 ------------------------------------------------------------------------------------------

D*.45.α ------------------------------------------------------------------------------------------

D*.45.αLYC ------------------------------------------------------------------------------------------

D*.66.α ------------------------------------------------------------------------------------------

D.66.α ------------------------------------------------------------------------------------------

C.39.28 361 LNPEAGMWQCLLSDSGQVLLESNIKVLPTWSTPVPRKIEVMYPPPYLDNEKSNGTIIHVKGKHLCPSPLFPGPSKP 433

D*.39.28 178 ----------------------------------.......................................... 247

D*.45.α 178 -------------------------------TTTPAPRPPTPAPTIASQPLSLRPEACRPAAGGAVHTRGLDFACD 251

D*.45.αLYC 178 -------------------------------TTTPAPRPPTPAPTIASQPLSLRPEACRPAAGGAVHTRGLDFACD 255

D*.66.α ----------ALSNSIMYFSHFVPVFLPAKPTTTPAPRPPTPAPTIASQPLSLRPEACRPAAGGAVHTRGLDFACD 270

D.66.α ----------ALSNSIMYFSHFVPVFLPAKPTTTPAPRPPTPAPTIASQPLSLRPEACRPAAGGAVHTRGLDFACD 265

C.39.28 434 FWVLVVVGGVLACYSLLVTVAFIIFWVRSKRSRLLCHSDYMNMTPRRPGPTRKHYQPYAPPRDFAAYRSKRGRKKLLYIFKQPFMRP 523

D*.39.28 248 ...................................-................................................... 336

D*.45.α 252 ---**IYIWAPLAGTCGVLLLSLVIT**---........-................................................... 335

D*.45.αLYC 256 ---**IYIWAPLAGTCGVLLLSLVIT**LYC........-................................................... 338

D*.66.α 271 ---**IYIWAPLAGTCGVLLLSLVIT**---........-................................................... 354

D.66.α 266 ---**IYIWAPLAGTCGVLLLSLVIT**---........-................................................... 349

C.39.28 524 VQTTQEEDGCSCRFPEEEEGGCELRVKFSRSADAPAYQQGQNQLYNELNLGRREEYDVLDKRRGRDPEMGGKPRRKNPQEGLYNELQKDK 613

D*.39.28 337 .......................................................................................... 426

D*.45.α 336 .......................................................................................... 425

D*.45.αLYC 339 .......................................................................................... 428

D*.66.α 355 .......................................................................................... 444

D.66.α 350 .......................................................................................... 439

C.39.28 614 MAEAYSEIGMKGERRRGKGHDGLYQGLSTATKDTYDALHMQALPPR 659

D*.39.28 427 .............................................. 472

D*.45.α 426 .............................................. 471

D*.45.αLYC 429 .............................................. 474

D*.66.α 445 .............................................. 490

D.66.α 440 .............................................. 485

https://services.healthtech.dtu.dk/services/TMHMM-2.0/

ALSNSIMYFSHFVPVFLPAKPTTTPAPRPPTPAPTIASQPLSLRPEACRPAAGGAVHTRGLDFACD **IYIWAPLAGTCGVLLLSLVITLY**C FWVRSKRSRLLCHSDYMNMTPRRPGPTRKHYQPYAPPRDFAAYRS

# WEBSEQUENCE Length: 135

# WEBSEQUENCE Number of predicted TMHs: 1

# WEBSEQUENCE Exp number of AAs in TMHs: 23.36174

# WEBSEQUENCE Exp number, first 60 AAs: 0.1579

# WEBSEQUENCE Total prob of N-in: 0.02453

WEBSEQUENCE TMHMM2.0 outside 1 66

WEBSEQUENCE TMHMM2.0 TMhelix 67 89

WEBSEQUENCE TMHMM2.0 inside 90 135
