# Supplementary material for: Extracellular domain, hinge, and transmembrane determinants affecting surface CD4 expression of a novel anti-HIV chimeric antigen receptor (CAR) construct
Source: PLoS One. 2024 Aug 12;19(8):e0293990. doi: 10.1371/journal.pone.0293990 (PMC11318886; doi:10.1371/journal.pone.0293990)
Supplement: S2 Table — (DOCX) [file pone.0293990.s006.docx]

**Supplemental Table S2**. Primer sequences used for construction of the different CARs using the splicing by overlap extension technique.

| **Splice Site** | **Sequence** |
| --- | --- |
| hCD8α hinge – hCD28 | F: AGGGGGCTGGACTTCGCCTGTGAT.TTTTGGGTGCTGGTGGTGGTTGGT |
|  | R: ACCAACCACCACCAGCACCCAAAAA.TCACAGGCGAAGTCCAGCCCCCT |
| hCD28 hinge – h4-1BB | F: ACTTCGCAGCCTATCGCTCC.AAACGGGGCAGAAAGAAACT |
|  | R: AGTTTCTTTCTGCCCCGTTTGGAGCGATAGGCTGCGAAGT |
| hCD28 – hTCRζ | F: GCAGCCTATCGCTCCAGAGTGAAGTTCAGCAGGAGCG |
|  | R: CCTGCTGAACTTCACTCTGGAGCGATAGGCTGCGAA |
| 5’ CAR | F: ATGATCGGATCCATGGCCTTACCAGTGACCGC |
| 3’ CAR | R: TCGACAGTCGACTTAGCGAGGGGGCAGGGCCT |
| 3’ truncated CAR | R: TCGACAGTCGACTTACGCGGGGGCGTCTGC |
